# Essential newborn care practices in health facilities of Nepal: Evidence from Nepal Health Facility Survey 2015 and 2021

**DOI:** 10.1371/journal.pgph.0002069

**Published:** 2024-04-25

**Authors:** Achyut Raj Pandey, Bikram Adhikari, Bipul Lamichhane, Bishnu Dulal, Saugat Pratap K. C., Deepak Joshi, Sushil Chandra Baral

**Affiliations:** HERD International, Kathmandu, Nepal; PLOS: Public Library of Science, UNITED STATES

## Abstract

Availability of newborn care practices in health facilities (HFs) plays an important role in improving the survival and well-being of newborns. In this paper, we aimed to assess practice of carrying out different newborn care practices among HFs between 2015 and 2021, and associated factors in Nepal. We analyzed data of 621 and 786 HFs offering delivery and newborn care services from Nepal Health Facility Surveys 2015 and 2021, respectively. We summarized categorical variables with a weighted percent and 95% confidence interval (CI). We estimated weighted unadjusted absolute difference in percentage of different newborn care practices between 2015 and 2021, and its 95% CI using binomial regression model. We applied univariable and multivariable logistic regression analysis to determine the factors associated with the availability of all seven newborn care practices. The percentage of HFs carrying out all seven newborn care practices was 50.5% (95% CI: 44.6, 56.3) in 2015 and 83.7% (95% CI: 79.8, 87.0) in 2021 with an overall difference of 33.3 percent points (95% CI: 26.4, 40.1). The proportion of HFs reporting all seven newborn care practices increased significantly between 2015 and 2021 in each all three ecological regions and in provinces except Madhesh and Gandaki. In 2021, private hospitals had lower odds of carrying out all seven newborn care practices compared to federal/provincial hospitals (AOR = 0.26, 95% CI: 0.11, 0.63). Similarly, in 2021, the odds of HFs carrying out all seven newborn care practices was 2.87 (95% CI: 1.06, 8.31) times higher in Sudurpashchim compared to Koshi province. In 2021, HFs carrying out seven newborn care practices did not differ significantly based on ecological belts, quality assurance activities, external supervision, delivery service-related training, and frequency of HF meetings. In conclusion, there has been significant improvement in proportion of facilities carrying out seven essential newborn care practices between 2015 and 2021. Type of facility and provinces were associated with the HFs carrying out seven newborn care practices in Nepal.

## Introduction

The first four weeks of life are the most important for a child’s survival [1]. Nearly 2.3 million children lost their lives in the first month of birth in 2021, which equates to about 6,400 deaths every day [2]. Sub-Saharan Africa and Central and Southern Asia have the highest mortality rates and the progress toward the worldwide 2030 goal varies at different rates in these nations [3]. From 1990 to 2020, the worldwide Neonatal Mortality Rate (NMR) fell by more than 50%, from 37 to 17 deaths per 1000 live births [2]. With a relatively slower pace of decline in NMR, the proportion of neonatal deaths out of total under-5 deaths increased from 39% in 2000 to 48% in 2019 [4,5].

While countries around the globe are struggling to reduce the NMR to 12 or fewer deaths per 1000 live births by 2030 as a part of Sustainable Development Goals (SDGs) [6], the alarming and avoidable burden of newborn deaths raises concerns about SDGs’ success as over 60 countries are predicted to fall short of the goal for newborn mortality [2]. If NMR continues to decline in the current rate, there will be 13.9 newborn fatalities per 1000 live births by 2030 [7], which is still more than the ideal NMR that can be achieved with the medical and technological advancements at hand [7]. According to an estimate based on predictions of the global burden of disease, the ideal worldwide NMR that could be achieved through medical and technological advancements is 0.80 deaths per 1000 live births [4].

In concordance with the global pattern, in Nepal, the decline in NMR has been relatively slow compared to the decline in under-5 mortality rates. Between 2001 to 2022, under-5 mortality rates declined from 91 per 1000 live births to 33 per 1000 live births in 2021, demonstrating a decline of approximately 64% in a period of two decades [8,9]. However, in the same period, NMR declined by 46% from 39 newborn deaths per 1000 live births in 2001 to 21 newborn deaths per 1000 live births in 2022 [8,9]. By achieving the SDG, Nepal will avoid 5,935 impairments and save an additional 27,116 neonatal lives [10]. Additionally, it has been demonstrated that programs aiming at improving maternal and newborn health are economically effective, with every dollar spent on newborn health yielding a return of $6 [10].

Newborns all over the world have the right to receive high-quality and essential newborn care which entails immediate medical attention after delivery and continued care throughout the newborn period at the health facility level as well as at home [11]. It includes immediate care at birth, thermal care, resuscitation when needed, support for breast milk feeding, nurturing care, infection prevention, assessment of health problems. Additionally, it involves recognition and response to danger signs, and timely and safe referral when needed. Immediate care at birth involves delayed cord clamping, thorough drying, assessment of breathing, skin-to-skin contact, and early initiation of breastfeeding. Essential newborn care is necessary both in the health facility and at home to ensure the well-being and survival of newborns [11]. While the progress on newborn health was challenged by the COVID-19 epidemic [3], the health system in Nepal underwent a transformation from unitary to a federal health system with substantial changes in health care delivery mechanism and the way health services are organized [12,13], which could pose additional challenges in health service delivery.

In Nepal, NMR has remained stagnant at 21 per 1000 live births between 2016 to 2022 despite improvements in service coverage indicators like antenatal care (ANC), health facility delivery, and postnatal care (PNC) visit [14]. Between 2016 to 2022, the proportion of pregnant women having 4 ANC visits increased from 71% to 81%, health facility delivery increased from 64% to 79%, and those receiving a PNC increased from 57% to 70% in the same period [14]. Between 2016 to 2022, the proportion of pregnant women having 4 ANC visits increased from 71% to 81%, health facility delivery increased from 64% to 79%, and those receiving a PNC check increased from 57% to 70% in the same period [14]. Stagnant NMR despite increasing coverage of pregnancy and delivery care raises concerns on the component of care they receive. In this context, we analyzed the change in availability of essential newborn care service from 2015 to 2021 and the variation by province, ecological belt and types of facilities. We have also identified the factors associated with essential newborn care service availability in 2015 and 2021.

## Methods

### Study design

We used data from two nationally representative Nepal Health Facility Survey (NHFS) conducted in the year 2015 and 2021. NHFS 2015 and NHFS 2021were implemented by NEW Era, a national research firm with the support from Ministry of Health and Population (MOHP) and technical support from ICF International.

Nepal’s healthcare system operates at three levels, federal, provincial, and local. According to public health regulation 2020, health services are provided by different level of health institutions at federal, provincial and local governments such as basic health service centers, basic hospitals (5–15 beds), general hospitals 25–50 bedded and 100–300 bedded), specialized hospitals, super specialty hospitals, teaching hospitals, children’s hospitals, basic ayurvedic service centers, ayurveda health centers (25–50 beds), specialized ayurveda hospitals, homeopathy hospitals (50 beds) and other health institutions (polyclinics, dental clinics etc.) [15].

The MoHP at federal level oversees policy formulation, planning, coordination, and organization of healthcare at all levels. The MoHP regulates services and implements healthcare initiatives. The local health system comprises various facilities such as basic hospitals, Primary health care centers (PHCCs) health posts, basic health service centres, urban health clinics, community health units, and community-level health facilities (HFs), including community health clinics, primary health outreach clinics, and immunization clinics. Health posts or basic health service centers serve as the initial point of contact for basic health services. Each local level is supposed to have a local hospital as per policy provision after the federalization process, which is currently being constructed [16,17]. Following the federalization process, as per policy provision, each local level is supposed to have a local hospital, which are currently being constructed [16,17]. Secondary and tertiary level care services are provided by provincial and federal level hospitals in Nepalese context. Each level higher than health posts serves as a referral point within a network that extends from PHCCs to primary and secondary level hospitals while private facilities, including hospitals, clinics, and pharmacies, deliver basic health services up to tertiary care.

### Sample and sampling techniques

In NHFS 2015, a total of 1000 HFs were selected out of 4719 eligible HFs using a random stratified sampling technique. A total of 963 healthcare facilities (HFs) were included in the study after eliminating 8 duplicate HFs and accounting for 29 HFs that did not participate due to refusal, closure, or inaccessibility caused by poor infrastructure. NHFS 2021 utilized a stratified random sampling method to select 1633 HFs from a pool of 5681 eligible facilities. Equal probability systematic sampling was employed, with sample allocation considering facility type within each province to achieve stratification. Out of the 1633 selected HFs, 97% (1576 facilities) successfully participated in the survey, while 2% were non-functional, 1% were unreachable, and 0.1% refused to take part in the study. In this study, we used data of 621 (weighted: 457) and 786 (weighted: 804) HFs offering normal vaginal delivery services from NHFS 2015 and NHFS 2021 respectively.

### Data collection

The NHFS 2015 data collection took place from April to November 2015, while the NHFS 2021 data collection occurred from January to September 2021. The data collection was carried out using four main data collection tools- a) facility inventory questionnaire, b) health provider interview questionnaire, c) observation for maternal and newborn care, and d) exit interview questionnaire. We used data from the facility inventory questionnaire focusing on newborn care practices in this study.

### Variables

#### Outcome variable

Those facilities reporting presence of all seven newborn care practices–delivery to the abdomen, drying and wrapping, kangaroo mother care (KMC), initiation of breastfeeding within the first hour, routine complete head-to-toe examination of the newborn before discharge, applying chlorhexidine gel to umbilical cord stump, and weighting the newborn immediately upon delivery were considered to have all seven newborn care practices.

#### Independent variables

The independent variables included ecological region (hill/mountain/terai), location of facility (rural/urban), province (Koshi / Madhesh / Bagmati / Gandaki /Lumbini / Karnali / Sudurpashchim), type of facility (federal/provincial hospital / local HFs / private hospital). The hospitals under federal or provincial government were classified as federal or provincial hospitals, facilities under local governments which include local hospitals, primary health care centers, and health posts were classified as local HFs, and the hospitals owned by private sectors were classified as private hospitals. For frequency of HFs meeting, the HFs stating “no” for routine management/administrative meetings were classified as “none”, those stating, “monthly or more often" were classified as “monthly” and those stating, “irregular or every 2–6 months” were classified as “sometimes”.

#### Statistical analysis

We used R program 4.2.0 for data cleaning and statistical analysis. We performed a weighted analysis to address complex survey design using the “survey” package. In the descriptive analysis, we summarized categorical variables with weighted percent and 95% confidence interval. We estimated the weighted unadjusted absolute difference in percentage of different newborn care practices between two year 2015 and 2021, and its 95% confidence interval using binomial regression model [18]. In inferential analysis, we applied univariate and multivariable logistic regression analysis to determine the association of availability of seven newborn care practices with the province, type of health facility and ecological region. The results of regression analysis were reported with crude and adjusted odds ratio along with their 95% CI and p-value. We considered a p-value less than 0.05 as statistically significant throughout this paper.

#### Ethical approval

The study involves a secondary analysis of NHFS 2015 and NHFS 2021. We obtained approval to use data for our research objective from “The DHS program”, (http://dhsprogram.com/data/available-datasets.cfm) [19]. In the original survey NHFS 2015 and 2021, ethical clearance was obtained from the Ethical Review Board of Nepal Health Research Council and ICF International.

## Results

**Fig 1:**
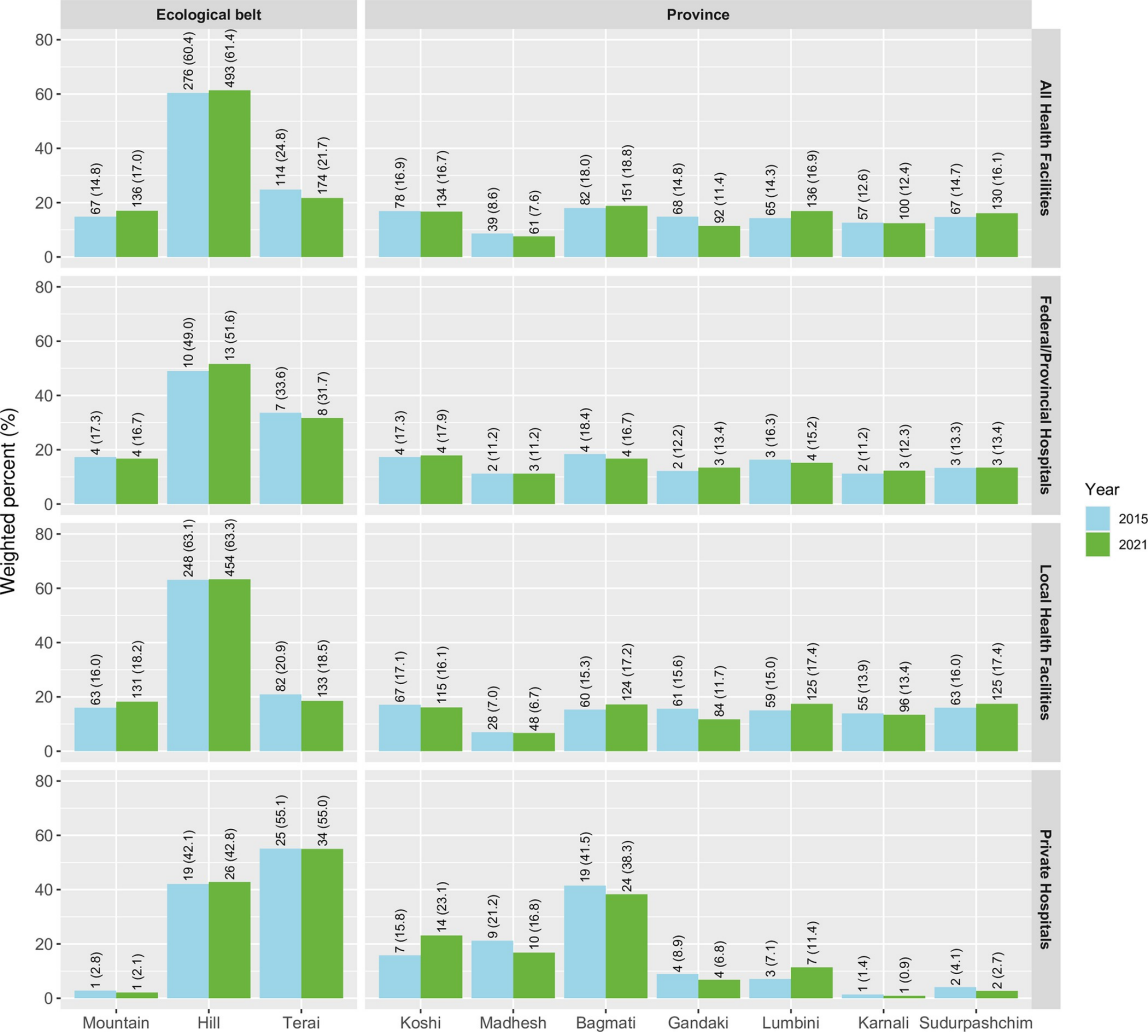
Distribution of facilities in NHFS 2015 and NHFS 2021.

The distribution of HFs offering normal vaginal delivery services by ecological belt and province between NHFS 2015 and 2021 were similar. In 2021, 61.4% facilities were from hill region, 17.0% facilities were from mountain region, and 21.7% facilities were from Terai region whereas in NHFS 2015, 60.4% facilities were from hill, 14.8% were from mountain and 24.8% were from Terai in 2015. In 2021, 16.7% of facilities were from Koshi, 7.6% were from Madhesh, 18.8% were from Bagmati, 11.4% were from Gandaki, 16.9% were from Lumbini, 12.4% were from Karnali and 16.1% were from Sudurpashchim. Fig 1 also shows distribution of HFs in NHFS 2015 and NHFS 2021. The representation of HFs between the two surveys looks comparable.

Table 1 presents the comparison of newborn care practices among HFs offering normal vaginal delivery services between NHFS 2015 and NHFS 2021. The proportion of HFs carrying out all seven newborn care practices was 50.5% (95% CI: 44.6, 56.3) in 2015 and 83.7% (95%CI: 79.8, 87.0) in 2021 with an overall increase of 33.3 percentage points (95%CI: 26.4, 40.1). In 2021, more than 90% facilities reported practicing delivery to the abdomen (96.1%, 95% CI: 93.7, 97.7), drying and wrapping of newborns (99.7%, 95% CI: 98.3, 99.9), early initiation of breastfeeding (99.4%, 95% CI: 98.1, 99.8), routine head-to-toe examination (97.4%, 95% CI: 95.3, 98.6), applying chlorhexidine gel to umbilical cord stump (96.5%, 95% CI: 94.9, 97.6) and weighing the newborn immediately upon delivery (99.0%, 95% CI: 96.8, 99.7).

**Table 1:** Comparison of availability of newborn care practices among HFs offering normal vaginal delivery between NHFS 2015 and NHFS 2021.

Newborn care practices	NHFS 2015	NHFS 2021	Change
	% (95% CI)	% (95% CI)	% (95% CI)
Delivery to the abdomen (skin to skin)	90.7 (86.5, 93.6)	96.1 (93.7, 97.7)	5.5 (1.5, 9.4)
Drying and wrapping newborns to keep warm	97.3 (94.4, 98.8)	99.7 (98.3, 99.9)	2.3 (0.2, 4.4)
Initiation of breastfeeding within the first hour	98.9 (96.6, 99.6)	99.4 (98.1, 99.8)	0.5 (-0.9, 2.0)
Routine complete (head-to-toe) examination of newborns before discharge	94.3 (90.7, 96.6)	97.4 (95.3, 98.6)	3.1 (-0.2, 6.4)
Applying chlorhexidine gel to umbilical cord stump	63.6 (57.8, 69.0)	96.5 (94.9, 97.6)	32.9 (27.2, 38.7)
KMC	91.4 (87.7, 94.1)	89.7 (86.2, 92.3)	-1.7 (-6.1, 2.6)
Weighing the newborn immediately upon delivery	95.5 (92.3, 97.5)	99.0 (96.8, 99.7)	3.5 (0.7, 6.2)
Vitamin K	9.8 (7.5, 12.7)	19.3 (15.9, 23.2)	9.4 (5.0, 13.9)
BCG	11.8 (8.8, 15.8)	10.0 (7.3, 13.5)	-1.8 (-6.4, 2.8)
Tetracycline eye ointment	12.9 (9.5, 17.4)	6.0 (4.1, 8.9)	-6.9 (-11.5, -2.3)
Availability of all 10 practices	0.2 (0.1, 0.6)	0.8 (0.4, 1.9)	0.6 (-0.1, 1.3)
Availability of first 7 practices	50.5 (44.6, 56.3)	83.7 (79.8, 87.0)	33.3 (26.4, 40.1

n: Weighted frequency; %: Weighted percent; CI: Confidence interval; BCG: Bacillus Calmette–Guérin.

The practice of applying chlorhexidine gel to umbilical cord stump increased from 63.6% (95% CI: 57.8, 69.0) in 2015 to 96.5% (95% CI: 94.9, 97.6) in 2021 with a total change of 32.9 percentage points (95% CI: 27.2, 38.7) and was the newborn care practice with the highest percentage point improvement. The practice of drying or wrapping newborns, routine head-to-toe examination of newborns, weighing newborns immediately after delivery and Vitamin K use improved positively between 2015 to 2021. There were no significant changes in the practices of initiation of breastfeeding within the first hour, Kangaroo Mother Care (KMC), and BCG vaccination between 2015 and 2021.

The details of the comparison of the facilities carrying out different newborn care practices in NHFS 2015 and NHFS 2021stratified by ecological belts, provinces and type of health facility is present in the **S1 Table**.

Disaggregated by type of facilities, use of Vitamin K and application of chlorhexidine were the two indicators that improved most between 2015 and 2021 in federal / provincial level hospitals. Use of Vitamin K increased from 20.5% (95% CI: 13.5, 29.8) in 2015 to 65.1% (95% CI: 54.5, 74.4) in 2021 demonstrating 44.6 percentage points improvement. Similarly, in local level facilities, the use of chlorhexidine was the indicator that improved most increasing from 67.6% (95% CI: 61.0, 73.5) to 98.7% (95% CI: 96.8, 99.4) demonstrating 31.1 percentage points improvement. In private hospitals, the proportion of facilities applying chlorhexidine gel to umbilical cord stump increased from 25.4% (95% CI: 16.2, 37.4) to 72.3% (95% CI: 62.4, 80.4) with a total of 46.9 percentage points improvement [Table 2].

**Table 2:** Newborn care practices by facility type.

Newborn care practices	Federal / provincial hospitals, % (95% CI)		Local level HFs, % (95% CI)		Private hospitals, % (95% CI)	
	2015, n = 20	2021. n = 25	2015, n = 392	2021, n = 718	2015, n = 45	2021, n = 61
Delivery to the abdomen (skin to skin)	87.8 (79.5, 93.0)	94.4 (87.0, 97.7)	91.5 (86.8, 94.7)	96.7 (94.0, 98.3)	84.4 (71.3, 92.2)	89.9 (79.4, 95.4)
Drying and wrapping newborns to keep warm	100.0	98.9 (92.2, 99.8)	97.2 (93.7, 98.8)	100.0	97.0 (85.0, 99.5)	95.9 (79.6, 99.3)
Initiation of breastfeeding within the first hour	99.0 (92.9, 99.9)	100.0	99.1 (96.1, 99.8)	99.7 (98.5, 99.9)	96.9 (85.0, 99.4)	95.3 (80.7, 99.0)
Routine complete(head-to-toe) examination of newborns before	99.0 (92.9, 99.9)	95.5 (88.5, 98.3)	94.2 (89.9, 96.7)	97.9 (95.4, 99.0)	94.0 (81.8, 98.2)	93.0 (81.2, 97.6)
Applying chlorhexidine gel to umbilical cord stump	70.4 (60.4, 78.7)	93.3 (85.7, 97.0)	67.6 (61.0, 73.5)	98.7 (96.8, 99.4)	25.4 (16.2, 37.4)	72.3 (62.4, 80.4)
KMC	94.8 (88.0, 97.9)	95.5 (88.4, 98.3)	92.4 (88.1, 95.2)	90.3 (86.5, 93.2)	81.6 (69.1, 89.8)	79.9 (70.0, 87.1)
Weighing the newborn immediately upon delivery	100.0	100.0	95.3 (91.6, 97.5)	99.2 (96.6, 99.8)	95.3 (82.7, 98.8)	96.4 (78.3, 99.5)
Vitamin K	20.5 (13.5, 29.8)	65.1 (54.5, 74.4)	2.4 (1.2, 4.7)	12.5 (9.2, 16.8)	70.4 (57.0, 80.9)	79.5 (69.6, 86.8)
BCG	31.7 (23.1, 41.7)	22.5 (14.9, 32.5)	9.7 (6.5, 14.2)	8.8 (6.0, 12.7)	22.0 (13.2, 34.2)	19.0 (11.1, 30.7)
Tetracycline Eye Ointment	15.3 (9.4, 24.0)	10.1 (5.3, 18.5)	14.0 (10.1, 19.1)	5.5 (3.3, 8.8)	2.9 (1.2, 6.5)	11.2 (7.1, 17.4)
Availability of all 10 practices	3.1 (1.0, 9.2)	2.2 (0.5, 8.7)	0.1 (0.0, 0.4)	0.7 (0.2, 2.0)	0.5 (0.1, 3.6)	1.7 (0.5, 5.3)
Availability of first 7 practices	60.2 (50.0, 69.5)	82.1 (72.5, 88.8)	53.4 (46.7, 59.9)	85.8 (81.4, 89.3)	20.5 (12.4, 31.9)	60.4 (50.2, 69.7)

n: Weighted frequency; %: Weighted percent; CI: Confidence interval; BCG: Bacillus Calmette–Guérin.

**Fig 2:**
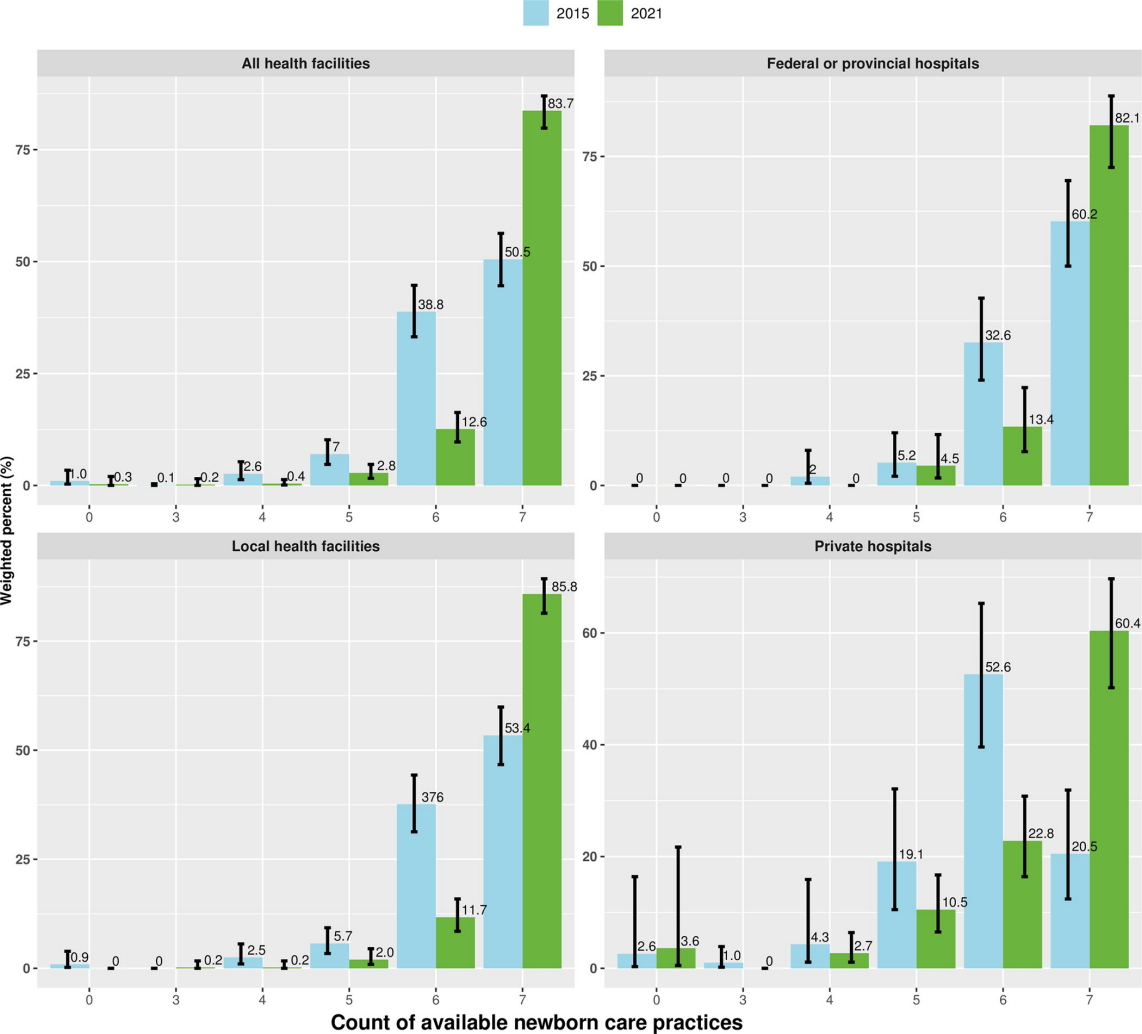
Comparison of availability newborn care practices score between NHFS 2015 and NHFS 2021.

Fig 2 shows the proportion of facilities reporting seven newborn care practices in terms of scores from one to seven. A score of one means that the facility carries out one out of seven newborn care practices while seven indicates that the facility carries out all seven newborn care practices. In 2021, 83.7% of HFs reported carrying out all seven newborn care practices which is an increase from 50.5% in 2015. Disaggregated by facility type, the proportion of facilities reporting all seven newborn care practices increased from 60.2% in 2015 to 82.1% in 2021 in federal or provincial level hospitals, 53.4% in 2015 to 85.8% in 2021 in local HFs and, from 20.5% in 2015 to 60.4% in 2021 in private hospitals.

Among ecological belts, the hill region witnessed the highest percentage point improvement in the practice of all seven newborn care practices. In the hill region, the proportion of facilities that reported practicing all seven newborn care practices increased from 46.6% (95% CI: 38.7, 54.7) in 2015 to 86.1% (95% CI:81.1, 89.9) in 2021 demonstrating a change of 39.5 percentage points (95% CI: 30.3, 48.6)). Among provinces, improvement in the newborn care practices was seen in Koshi, Bagmati, Lumbini, Karnali and Sudurpashchim whereas there was no significant improvement in Madhesh and Gandaki. In Karnali province, percentage of facilities with all newborn care practices increased from 33.0% (95% CI: 19.0,50.7) in 2015 to 85.9% (95% CI: 74.9, 92.5) in 2021 demonstrating a change of 52.9 percentage points (95% CI: 34.8, 71.0). Similarly, in Bagmati province, the percentage of facilities with all seven newborn care practices improved from 36.9% (95% CI: 25.7, 50.0) in 2015 to 89.7% (95% CI: 80.6, 94.8) in 2021 demonstrating a change of 52.8 percentage points (95% CI: 38.7, 66.8). All three levels of facilities, federal/provincial, local HFs and private hospitals showed improvement in proportion of facilities with availability of all seven newborn care practices between 2015 to 2021 [Table 3].

**Table 3:** Comparison of availability of all seven newborn care practices between 2015 and 2021 by ecological region, province, and type of facility.

**Characteristics**	**NHFS 2015**		**NHFS 2021**		**Change, 95% CI**
	**n**	**% (95%CI)**	**n**	**% (95%CI)**	
**Ecological region**					
Hill	276	46.6 (38.7, 54.7)	493	86.1 (81.1, 89.9)	39.5 (30.3, 48.6)
Mountain	68	53.5 (40.4, 66.2)	136	83.2 (71.6, 90.7)	29.7 (13.6, 45.7)
Terai	114	58.0 (47.8, 67.5)	174	77.5 (68.0, 84.8)	19.5 (6.5, 32.5)
**Province**					
Koshi	78	53.6 (39.9,66.7)	134	76.6 (64.4, 85.6)	23.1 (5.8, 40.3)
Madhesh	39	58.3 (40.5,74.1)	61	74.7 (55.1, 87.7)	16.4 (-7.3, 40.2)
Bagmati	82	36.9 (25.7, 50.0)	151	89.7 (80.6, 94.8)	52.8 (38.7, 66.8)
Gandaki	68	50.2 (32.5, 67.9)	92	68.1 (52.5, 80.5)	17.9 (-5.2, 40.9)
Lumbini	65	62.2 (47.5, 74.9)	136	89.3 (79.3, 94.8)	27.2 (11.4, 42.9)
Karnali	57	33.0 (19.0,50.7)	100	85.9 (74.9, 92.5)	52.9 (34.8, 71.0)
Sudurpashchim	67	62.6 (48.2,75.1)	130	91.9 (83.6, 96.2)	29.3 (14.4, 44.1)
**Type of facility**					
Federal/provincial hospitals	20	60.2 (50.1,69.5)	25	82.1(72.6, 88.8)	21.9 (9.4, 34.5)
Local level HFs	392	53.4 (46.7,59.9)	718	85.8 (81.4, 89.3)	32.4 (24.7, 40.1)
Private hospitals	45	20.5 (12.5,31.8)	61	60.4 (50.2, 69.7)	39.9 (26.2, 53.6)

n: Weighted frequency; %: Weighted percent; CI: Confidence interval.

In multivariate analysis presented in Table 4, the odds of practicing all newborn care practices in the year 2015 was 0.17 (AOR: 0.17, 95% CI: 0.07, 0.04) times in private hospitals compared with federal/provincial hospitals, 2.45 (AOR: 2.45, 95% CI: 1.04, 5.76) times in HFs having health facility meeting sometimes and 2.1 (AOR: 2.10, 95% CI: 1.03, 7.99) times in HFs having monthly health facility meeting compared to HFs with no health facilities meeting. In the year 2021, the odds of practicing all seven newborn care practices was 2.87 (AOR: 2.87, 95% CI: 1.03, 7.99) times higher in Sudurpashchim province compared to Koshi. Similarly, the odds of having all seven newborn care practices was 0.26 (AOR: 0.26, 95% CI: 0.11, 0.63) times in private hospitals compared to federal/provincial hospitals.

**Table 4:** Association of factors with availability of all seven newborn care practices.

Characteristics	NHFS 2015				NHFS 2021			
	Unadjusted		Adjusted		Unadjusted		Adjusted	
	OR (95%CI)	p-value	AOR (95% CI)	p-value	OR (95%CI)	p-value	AOR (95% CI)	p-value
**Ecological Region**								
Hill	Ref		Ref		Ref		Ref	
Mountain	1.32 (0.71, 2.44)	0.378	1.45 (0.75, 2.79)	0.266	0.80 (0.37, 1.72)	0.572	0.64 (0.26, 1.54)	0.316
Terai	1.58 (0.94, 2.66)	0.086	1.55 (0.78, 3.07)	0.213	0.56 (0.30, 1.02)	0.058	0.59 (0.26, 1.34)	0.210
**Province**								
Koshi	Ref		Ref		Ref		Ref	
Madhesh	1.21 (0.49, 2.96)	0.678	0.96 (0.35, 2.68)	0.945	0.90 (0.32, 2.57)	0.843	1.20 (0.37, 3.92)	0.763
Bagmati	0.51 (0.24, 1.09)	0.081	0.56 (0.24, 1.31)	0.183	**2.65 (1.03, 6.79)**	**0.042**	2.53 (0.93, 6.90)	0.069
Gandaki	0.87 (0.35, 2.17)	0.771	0.88 (0.33, 2.35)	0.804	0.65 (0.27, 1.57)	0.336	0.46 (0.18, 1.22)	0.119
Lumbini	1.42 (0.64, 3.18)	0.391	1.41 (0.59, 3.39)	0.441	**2.54 (0.96, 6.73)**	**0.060**	2.22 (0.80, 6.14)	0.125
Karnali	0.43 (0.17, 1.06)	0.065	0.41 (0.16, 1.03)	0.058	1.85 (0.74, 4.64)	0.187	1.42 (0.52, 3.92)	0.497
Sudurpashchim	1.45 (0.65, 3.22)	0.362	1.26 (0.55, 2.87)	0.586	**3.45 (1.28, 9.29)**	**0.014**	**2.87 (1.03, 7.99)**	**0.044**
**Type of Facility**								
Federal/provincial hospital	Ref		Ref		Ref		Ref	
Local level facilities	0.76 (0.47, 1.23)	0.263	0.81 (0.47, 1.42)	0.469	1.32 (0.70, 2.48)	0.393	1.09 (0.53, 2.26)	0.811
Private hospital	**0.17 (0.08, 0.35)**	**<0.001**	**0.17 (0.07, 0.40)**	**<0.001**	**0.33 (0.17, 0.66)**	**0.002**	**0.26 (0.11, 0.63)**	**0.003**
**Quality assurance activities**								
Not Performed	Ref		Ref		Ref		Ref	
Performed	0.70 (0.42, 1.19)	0.189	0.57 (0.31, 1.03)	0.062	1.10 (0.61, 1.98)	0.75	1.01 (0.52, 1.94)	0.983
**External supervision**								
No	Ref		Ref		Ref		Ref	
Yes	1.41 (0.83, 2.41)	0.207	1.15 (0.65, 2.05)	0.626	1.55 (0.90, 2.68)	0.112	1.42 (0.78, 2.57)	0.248
**Provider with training on delivery**								
No staff with training	Ref		Ref		Ref		Ref	
At least one staff with training	**1.78 (1.08, 2.91)**	**0.023**	1.40 (0.83, 2.36)	0.207	0.88 (0.48, 1.60)	0.666	0.69 (0.35, 1.36)	0.285
**Health facility meeting frequency**								
None	Ref		Ref		Ref		Ref	
Sometimes	**2.28 (1.00, 5.18)**	**0.049**	**2.45 (1.04, 5.76)**	**0.040**	1.53 (0.59, 3.98)	0.379	1.39 (0.50, 3.87)	0.533
Monthly	**2.21 (1.19, 4.09)**	**0.012**	**2.10 (1.09, 4.04)**	**0.026**	1.44 (0.65, 3.19)	0.371	1.36 (0.58, 3.21)	0.477

OR = Odds Ratio, AOR = Odds Ratio, CI = Confidence Interval; Ref: Reference group.

Bold indicates significance at 95% confidence interval.

## Discussion

In this further analysis of NHFS 2015 and 2021, we determined change in proportion of facilities practicing seven newborn care practices and factors associated with it in 2015 and 2021. The proportion of facilities practicing seven newborn care practices has increased between 2015 and 2021.

In 2021, the proportion of HFs with practicing of delivery to abdomen service was 96.1%, drying and wrapping newborns to keep warm was 99.7%, early initiation of breast feeding within first hour of birth was 99.4% and routine complete (head to toe) examination of newborn before discharge was 97.4%. Similarly, the proportion of HFs applying chlorohexidine gel in umbilical cord stump was 96.5% and those with administration of Vitamin K was 19.3%. These findings are similar to the findings reported in Bangladesh from health facility survey in the year 2017 [20]. Health facility Survey in Bangladesh reveals that the proportion of HFs practicing delivery to the abdomen is 87.5% in facilities (excluding community clinics), drying and wrapping newborns to keep warm was 96.2%, initiating breastfeeding within one hour was 99%, routine complete (head to toe) examination of newborn before discharge was 91.3%, application of Vitamin K administration was 35.8%, and application of 7.1% chlorhexidine was 82.9% [20].

In 2021, private hospitals had lower odds of engaging in seven newborn care practices compared to federal/provincial hospitals. Sudurpashchim province had almost 3-fold higher odds of practicing all seven newborn care practices compared to Koshi province. This could be because Sudurpashchim province has placed higher priority in health sector compared to other provinces, which could have led to higher investment, enhanced quality control initiatives and thus better practices. For example, the per capita spending on health increased from 607 per capita to 2941 per capita in Sudurpashchim province demonstrating approximately 3.8 fold increase while it increased from 1821 to 3432 at the national level which reflects around 88% increase in the same period [8].

The proportion of HFs practicing all seven newborn care practices increased from 51% to 84% with almost 33 percentage points increase. This progress has been driven by an increase in coverage of increase in the proportion of HFs applying chlorhexidine gel to umbilical cord stump which increased from 64% to 97%, with almost 33 percentage points increase in the period. Based on the assessment conducted in 10 districts, the government of Nepal (GoN) decided to scale up the chlorhexidine programme to 41 districts by 2014 [21]. In the first phase of chlorhexidine programme between October 2011 and September 2014, application of chlorhexidine were implemented in 49 districts which were then gradually scaled up throughout the country after October 2014 [22]. By Mid of 2016, 58 districts were implementing chlorhexidine at health facility and community levels by mid-2016 which has not been scaled up throughout the country [21,23]. The jump in the proportion of HFs applying chlorhexidine gel to the umbilical cord stump could be because of policy changes or the decision of the GoN to scale up the programme throughout the country.

Despite the high importance placed on the KMC, the proportion of HFs practicing KMC declined from 91% to 90% between 2015 to 2021. The KMC approach encompasses several interventions, such as continuous and early skin-to-skin contact, assistance with breastfeeding, early hospital discharge, and supportive care for stable neonates. Implementing this care package in hospitals for preterm neonates weighing less than 2000 g has been identified to decrease the risk of neonatal mortality by 51% [24], hypothermia by 77% [25], and can shorten the duration of hospital stay. Although 91% of facilities reported adopting KMC, the coverage seems to decline further when it is captured at population level as all newborns delivered or visiting facilities with KMC may not receive the service. Multiple Indicators Cluster Survey in 2019 reported that the proportion of babies receiving KMC service was a cost-effective intervention to care for stable preterm/low birth weight (LBW) babies that is being implemented by the GoN as special care for small and/or sick new-born. Although skin–to-skin contact has been part of different programs/ training packages such as Community-Based Integrated Management of Neonatal and Childhood Illnesses (CB-IMNCI), Facility-Based Integrated Management of Neonatal and Childhood Illnesses (FB-IMNCI), Skilled Birth Attendants (SBA) training and Comprehensive level-II new-born care, a full-fledged KMC program was rolled out in the year 2021 [21].

Despite 33 percentage point increment in proportion of HFs practicing seven signal functions available, the latest evidence from Nepal Demographic and Health Survey (NDHS) 2022 shows that NMR has remained stagnant at 21 per 100,000 live births between 2016 to 2021[8,23]. As discussed above, this might be because chlorhexidine has substantial contribution in driving the aggregate figures upward which has limited contribution in reducing the NMR in Nepalese context. There is evidence with moderate certainty that using chlorhexidine on the umbilical cord stump as a routine practice does not appear to significantly impact neonatal mortality when compared to dry cord care or usual care [26]. World Health Organization has set context specific recommendations regarding use of chlorhexidine, recommending only in setting where harmful traditional substances (e.g. animal dung) are commonly used on the umbilical cord [26].

Our study suggests that a relatively lower proportion of private hospitals tend to practice all seven signal functions for newborns compared to federal/provincial hospitals. Private hospitals have important role in maternal and newborn health service delivery in Nepal. For example, as per NDHS 2016, out of total 57% deliveries take place in HFs; 10.2% deliveries take place in private facilities. Similarly, out of 57% newborns who received first PNC checkup within 2 days of birth, 41% had received such service from government while the contribution of private sector was 10% which indicate the need of involving private sector in expanding quality newborn service coverage and improving neonatal health outcomes [23]. However, low proportion of private hospitals practicing all seven newborn care practices has raised concerns on compliance to standard protocol and quality of service. Government should set strategies to regularly monitor and supervise private HFs relating to newborn service delivery. Steps may be taken to enhance the capacity of private HFs through organizational twining, whereby, public hospitals with better compliance to newborn care practices and yielding better health outcomes may coach or engage with selected private hospital within the province to improve compliance and quality of care. Regular interaction with private sector service providers, periodic discussion on policy provisions and facilitating them to develop infrastructures required for newborn services could be useful. Further studies, particularly exploring which factors or characteristics of the provinces are linked to better or poor newborn care practices could be useful. Further, strategies particularly tailored to the context of provinces having poor newborn care practices could be beneficial.

There are several strengths of this study. First, data collection for NHFS 2015 and NHFS 2021 was carried out in nationally representative samples, so the finding of this study is generalizable to Nepalese context. Second, NHFS 2015 and 2021 used validated demographic and health survey tools and the determination of availability of newborn care practices are based on the observation by the enumerator thus ensuring the validity. Third, it is the first study based on nationally representative sample of HFs that allows us to compare the progress in newborn care practices after Nepalese health care delivery system underwent federalization process.

Despite several strengths, there are some limitations to this study. First, we utilized data from a NHFS 2021 that was designed to assess the service availability and readiness for multiple services like antennal care, family planning, non-communicable diseases related services and so on and was not primarily dedicated to essential newborn care so some important variables like number of deliveries, newborn complications that could provide better context to the findings are missing. Second, the data collection of NHFS 2021 was undertaken during the period of COVID-19 pandemic, newborn care practices may have been under-estimated or over-estimated in NHFS 2021 owing to changes in service organization during the pandemic period. Third we have used the same weight for all newborn care practices to define availability of all seven newborn care practices. However, they may have different levels of impact or importance in improving neonatal health outcome or preventing morbidity or mortality.

## Conclusion

The availability of seven newborn care practices has increased between 2015 and 2021 and was relatively lower in private hospitals compared to federal or provincial hospitals. Further scaling up and quality improvement of newborn care practices by HFs is essential to improve newborn survival and well-being.

## Supporting information

S1 Table Availability of all seven newborn care practices by facility type. (DOCX)
