# Supplementary material for: Essential newborn care practices in health facilities of Nepal: Evidence from Nepal Health Facility Survey 2015 and 2021
Source: PLOS Glob Public Health. 2024 Apr 25;4(4):e0002069. doi: 10.1371/journal.pgph.0002069 (PMC11045114; doi:10.1371/journal.pgph.0002069)
Supplement: S1 Table — (DOCX) [file pgph.0002069.s001.docx]

S1 Table: Availability of all seven newborn care practices by facility type

|  | **Federal/provincial** | | **Local** | | **Private** | |
| --- | --- | --- | --- | --- | --- | --- |
|  | **2015** | **2021** | **2015** | **2021** | **2015** | **2021** |
|  | **% (95% CI)** | **% (95% CI)** | **% (95% CI)** | **% (95% CI)** | **% (95% CI)** | **% (95% CI)** |
| **Ecological belt** |  |  |  |  |  |  |
| Hill | 58.2  43.6, 71.6) | 84.9  (70.9, 92.8) | 48.4  (39.6, 57.3) | 87.3  (81.9, 91.3) | 17.1  (7.5, 34.5) | 65.1  (46.4, 80.1) |
| Mountain | 64.7  (38.3, 84.4) | 86.7  (55.8, 97.1) | 53.3  (39.3, 66.8) | 83.2  (71.0, 90.9) | 33.3  (5.1, 82.4) | 78.6  (8.2, 99.3) |
| Terai | 60.6  (42.6, 76.1) | 75.1  (55.1, 88.2) | 68.4  (55.0, 79.4) | 83.1  (69.9, 91.3) | 22.4  (11.2, 39.9) | 56  (44.1, 67.2) |
| **Province** |  |  |  |  |  |  |
| Koshi | 52.9  (28.5, 76.0) | 68.8  (40.9, 87.5) | 58  (42.4, 72.2) | 81.7  (66.7, 90.9) | 11.7  (3.3, 33.7) | 38.2  (23.2, 55.8) |
| Madhesh | 63.6  (30.1, 87.7) | 80  (39.8, 96.0) | 70.8  (48.7, 86.1) | 79.9  (52.3, 93.5) | 20.3  (4.1, 60.0) | 49  (30.4, 67.8) |
| Bagmati | 55.3  (31.2, 77.2) | 80  (49.8, 94.2) | 41.6  (26.3, 58.7) | 92.6  (80.8, 97.4) | 18.1  (7.1, 38.9) | 76.2  (53.3, 90.0) |
| Gandaki | 41.7  (16.4, 72.3) | 83.3  (47.4, 96.5) | 52.4  (32.8, 71.4) | 68.7  (51.5, 81.9) | 21.6  (2.9, 71.6) | 43.3  (18.9, 71.4) |
| Lumbini | 68.8  (40.9, 87.5) | 92.3  (55.0, 99.2) | 62.8  (46.6, 76.6) | 90.3  (78.9, 95.8) | 43  (13.0, 79.2) | 70.4  (45.7, 87.0) |
| Karnali | 81.8  (43.9, 96.3) | 81.8  (43.8, 96.3) | 31.3  (17.0, 50.4) | 85.9  (74.5, 92.7) | - | 100 |
| Sudurpaschim | 61.5  (31.5, 84.8) | 91.7  (52.1, 99.1) | 63.2  (47.6, 76.4) | 92  (83.2, 96.4) | 44.4  (14.5, 79.1) | 83.3  (22.9, 98.8) |
